# Impact of root hairs on microscale soil physical properties in the field

**DOI:** 10.1007/s11104-022-05530-1

**Published:** 2022-06-11

**Authors:** M. Marin, P. D. Hallett, D. S. Feeney, L. K. Brown, M. Naveed, N. Koebernick, S. Ruiz, A. G. Bengough, T. Roose, T. S. George

**Affiliations:** 1 School of Biological Sciences, University of Aberdeen, Aberdeen, AB24 3UU UK; 2 The James Hutton Institute, Invergowrie, Dundee, DD2 5DA UK; 3 School of Science and Engineering, University of Dundee, Dundee, DD1 4HN UK; 4 Present Address: School of Computing and Engineering, University of West London, London, W5 5RF UK; 5 Faculty of Engineering and Physical Sciences, University of Southampton, Southampton, SO17 1BJ UK; 6 Present Address: Institute of Agricultural and Nutritional Sciences, Martin Luther University Halle-Wittenberg, 06108 Halle (Saale), Germany

**Keywords:** Barley, Root hairs, Soil health, Soil hydromechanical properties, Soil structure, Soil water retention

## Abstract

**Aims:**

Recent laboratory studies revealed that root hairs may alter soil physical behaviour, influencing soil porosity and water retention on the small scale. However, the results are not consistent, and it is not known if structural changes at the small-scale have impacts at larger scales. Therefore, we evaluated the potential effects of root hairs on soil hydro-mechanical properties in the field using rhizosphere-scale physical measurements.

**Methods:**

Changes in soil water retention properties as well as mechanical and hydraulic characteristics were monitored in both silt loam and sandy loam soils. Measurements were taken from plant establishment to harvesting in field trials, comparing three barley genotypes representing distinct phenotypic categories in relation to root hair length. Soil hardness and elasticity were measured using a 3-mm-diameter spherical indenter, while water sorptivity and repellency were measured using a miniaturized infiltrometer with a 0.4-mm tip radius.

**Results:**

Over the growing season, plants induced changes in the soil water retention properties, with the plant available water increasing by 21%. Both soil hardness (*P* = 0.031) and elasticity (*P* = 0.048) decreased significantly in the presence of root hairs in silt loam soil, by 50% and 36%, respectively. Root hairs also led to significantly smaller water repellency (*P* = 0.007) in sandy loam soil vegetated with the hairy genotype (-49%) compared to the hairless mutant.

**Conclusions:**

Breeding of cash crops for improved soil conditions could be achieved by selecting root phenotypes that ameliorate soil physical properties and therefore contribute to increased soil health.

**Supplementary Information:**

The online version contains supplementary material available at 10.1007/s11104-022-05530-1.

## Introduction

Plants manipulate soil structure and hydrology through root growth and functional traits such as root branching and penetration of strong soils (Benard et al. 2016; Bengough 2012; Carminati et al. 2010; Gregory et al. 2010; Tisdall and Oades 1979). As a root penetrates soil, the action of root hairs and mucilage exudation at the root-soil interface constantly manipulates soil structure, influencing particle aggregation, porosity, and water retention (Bengough 2012; Hinsinger et al. 2009; Mueller et al. 2019). The volume of soil directly influenced by living roots represents the rhizosphere (Hinsinger et al. 2009). In shallow soils cultivated with cereal crops, the volume of the rhizosphere can account for up to half of the soil volume (Bengough 2012). However, rhizosphere volume can vary markedly, depending on the properties of the soil and plant-driven processes such as water depletion, extracted nutrients or released exudates (Schlüter et al. 2018).

The rhizosphere represents the most hydrologically active region of the soil, mediating all plant water uptake from soil, and hence the transpiration process (Bengough 2012). The change in soil porosity with root growth has been reported to be species-dependent, with coarse root systems increasing macroporosity by 30% and species with dense fine root systems enhancing mesopore volume (Bodner et al. 2014). However, literature data on rhizosphere porosity has also shown contrasting results, with some studies showing decreased rhizosphere porosity. For instance, Bruand et al. (1996) reported a densification of soil close to maize roots (23% less pore space). Root-induced compaction, with consequent increase in contact areas between loosely packed aggregates and therefore greater unsaturated hydraulic conductivity of the rhizosphere soil was also found by Aravena et al. (2011).

In addition to affecting porosity, roots modify the rhizosphere hydraulic properties by exuding mucilage, which absorbs water and therefore increases the water content at a given matric potential (Carminati et al. 2010; McCully and Boyer 1997), while also lowering the surface tension of pore water, sometimes with exudates that turn hydrophobic upon drying onto soil particles (Moradi et al. 2011; Read et al. 2003). Indeed, imaging techniques showed that the soil next to the roots remained wetter than the bulk soil under drying around roots of *Lupinus angustifolius* (Garrigues et al. 2006), *Pinus taeda* (MacFall et al. 1990) and *Hordeum vulgare* (Segal 2008), while after rewetting the rhizosphere remained relatively dry in the proximity of roots in *Cicer arietinum*, *Lupinus albus* and *Zea mays* (Carminati et al. 2010; Moradi et al. 2011; Tumlinson et al. 2008). Hallett et al. (2003) directly measured the hydraulic properties of the rhizosphere and found reduced water sorptivity in moist rhizosphere soil of barley compared with bulk soil due to increased water repellency in the rhizosphere. Zarebanadkouki et al. (2018) showed that water repellency of the rhizosphere reduced root water uptake by a factor of 6.5 times during the first 2–3 h after irrigation.

Laboratory studies demonstrated that specific root traits associated with the rhizosphere, such as exudate composition and root hairs, influence soil hydro-mechanical properties (Naveed et al. 2018; Read et al. 2003) and plant uptake of water and nutrients (Carminati et al. 2017; George et al. 2014). Treating sandy and clay loam textured soils with root exudates and exudate analogues (chia-seed mucilage) was found to enhance water repellency and mechanical hardness (Naveed et al. 2018). Microbial and fungal communities (*e.g*., mycorrhizal hyphae) associated with rhizosphere soil can also influence soil porosity, aggregation and stability (Caravaca et al. 2005; Feeney et al. 2006). However, root hairs have been observed to be the main agents affecting soil stability in the rhizosphere (Moreno-Espíndola et al. 2007). Koebernick et al. (2017, 2019) investigated the effect of root hairs on soil porosity and pore connectivity, comparing the wildtype and a hairless barley mutant in an extremely controlled environment (4.2 mm soil columns imaged by synchrotron tomography). While Koebernick et al. (2017) found that the wildtype genotype with root hairs increased soil macroporosity in the rhizosphere compared to the hairless mutant, Koebernick et al. (2019) did not find any effect of root hairs on pore structure in the rhizosphere. Therefore, although it is known that root hairs can influence rhizosphere soil structure on the small scale (*i.e*., laboratory experiments), the results are not consistent, and it is not known if structural changes at the small-scale have impacts at larger scale (*i.e*., open field) where soil heterogeneity may have major effects on root-soil interactions (Phalempin et al. 2021).

Field studies on the effects of “rhizosphere traits” (*e.g*., root hairs and mucilage) are severely lacking. A recent study highlighted the effect of root hairs for plant water status (*i.e*., drought-tolerance) and yield stability under a real agricultural scenario (*i.e*., open field over two contrasting seasons; Marin et al. (2021)). However, this study did not investigate the potential effects of root hairs on soil physical properties, which would benefit agricultural systems. Therefore, the present study extends previous research of Marin et al. (2021) by including a soil perspective on the potential benefits of root hairs. To the best of our knowledge only Phalempin et al. (2021) conducted a detailed study (laboratory and field experiments) on the factors affecting soil structure around roots. However, this study mainly focussed on rhizosphere scale bulk density alterations and no effect of root hairs was detected. In the present study, the potential effects of root hairs on spatial and temporal variability of the hydro-mechanical properties of bulk soil are evaluated from plant establishment to harvesting in a real agricultural landscape.

We assessed the influence of root hairs on soil water retention, pore structure and mechanical properties to address the following hypotheses: 1) root hairs actively restructure soil pores in field conditions, resulting in increased soil water retention and water absorption capacity; 2) the presence of root hairs drives mechanical and hydrological changes in the soil under field conditions; 3) plant-soil interactions affect soil physical properties over a growing season, improving the conditions for plant growth; 4) crop breeding could select root traits to improve soil physical conditions. The hypotheses were tested in a full-scale field experiment using barley genotypes exhibiting variations in root hair length and density. If root hairs do influence soil structure beyond the rhizosphere, this trait could have benefits to soil sustainability that could be targeted in plant breeding.

## Materials and methods

### Field experiment

The field experiment was carried out at The James Hutton Institute, Dundee, UK (56°27′34·80″ N, 3°4′21·01″ W) at the top and bottom a gently sloping field to obtain different soil textures: sandy loam (59% sand, 37% silt and 4% clay; Dystric Cambisol) and silt loam (47% sand, 48% silt and 5% clay; Haplic Cambisol; Naveed et al. (2018)). Clays in this field are predominantly 2:1 with traces of kaolinite (Barré and Hallett 2009). The soil texture analysis was carried out on samples collected at two soil depths (0–13 cm and 14–27 cm) on three replicates per location, for a total of 12 samples (Suppl. Figure 1). The USDA soil classification system was used for texture analysis, which was determined with the combination of wet sieving and laser diffraction after sonicating for 5 min (for particles smaller than 0.250 mm; Mastersizer 2000, Malvern Instruments, UK). Three barley (*Hordeum vulgare*) genotypes representing distinct phenotypic categories in relation to root hair length were used in the study. These included the cv Sassy with abundant root hairs and cv Optic, which is the wildtype (WT) to a no root hair line (NRH) derived from an ethylmethane sulfonate (EMS) mutant barley population (data on root hair length and density can be found in Marin et al. (2021)). Seeds were sown at 4 cm depth on 25/4/2018 in each of the sandy loam and silt loam fields. Plots containing each genotype were 1.5 × 6 m and were replicated four times following a random block design (for more details see Marin et al. (2021)).

### Root biomass and root length

During the growing season whole plants were harvested at different time points and their root length and biomass were assessed. Two plants per plot were sampled at four time points in 2018: 19 (May 14), 33 (May 28), 49 (June 13) and 61 (June 25) days after sowing (DAS). Roots of individual plants were washed with water, spread out and floated in a small amount of water in a standard petri dish and placed against a white background. An image was taken in greyscale (600 dpi) using an Epson Expression 10000XL scanner (Epson UK, London). The software WinRHIZO pro (Regent Instruments, Quebec City, Canada) was used to digitally map root samples and calculate root length for the following root diameter classes: 0–0.4 mm; 0.4–1 mm; 1–1.5 mm; 1.5–2 mm; > 2 mm. Root biomass was measured by weighing oven-dried material, which had been dried at 70 °C for 4 days.

### Soil water retention

Intact soil cores (55 mm diameter and 45 mm height) were sampled during the growing season (May – August) at 0.1 m (0.05 – 0.10 m) and 0.2 m (0.15 – 0.20 m) depth in each treatment plot and used to measure soil water content (*θ*) at saturation, at about field capacity (*θ* at -5 kPa) and at the permanent wilting point (PWP, *θ* at -1500 kPa). In each of the silt and sandy loam soils, cores were sampled three times: at 19 – 20 (May 14 – 15), 50 – 51 (June 14 – 15) and 124 (August 27) DAS. The soil water content was determined using a ceramic suction plate at -5 kPa controlled by a bubbling tower and vacuum pump (Soilmoisture Equipment, USA), and a pressure plate apparatus at -1500 kPa (Soilmoisture Equipment, USA). During testing, pressure was applied to the soil cores placed on the ceramic plate to extract water until the soil water content reached an equilibrium with the applied pressure. At equilibrium, water content (*θ*) was stable and could be determined by weighing the soil sample (Bittelli 2010; Smith 2000). For each time point, soil texture and genotype, four replicates were sampled at 0.1 and 0.2 m depths, for a total of 144 soil cores that were tested for soil water retention properties. Data were used to calculate the plant available water (PAW, *i.e.,* difference between *θ* at -5 kPa and *θ* at -1500 kPa) and air capacity (*i.e*., difference between *θ* at saturation and *θ* at field capacity). Pore volume for different pore diameter classes (aeration pores (> 300 µm); drainage pores (300 – 30 µm); slow drainage and retention pores (30 – 0.2 µm); pores holding inaccessible water for plants (< 0.2 µm)) and porosity (*f*) were estimated from the soil water retention data. The effective pore diameter (*d*) corresponding to the water potential (*Ψ*) tested in the SWRCs was calculated using Jurin's formula (Eq. 1; Rousseva et al. (2017)).

1 $$d=4\times \frac{\sigma }{\Psi }$$

where the surface tension is σ = 7.29 × 10^–2^ N m^−1^ and *Ψ* is in Pa. For instance, the effective diameter of a pore corresponding to 1500 kPa is 0.2 μm. Pore volume for the different pore diameter classes was expressed per core volume (m^3^ m^−3^).

### Soil mechanical properties

During the soil water retention measurements, soil penetration resistance (PR; MPa) was determined on samples equilibrated at -5 and -20 kPa for a total of 288 tests. Soil resistance to penetration was determined using a universal testing frame (Instron 5966, Norwood, MA, USA) fitted with a 50 N loading cell. In each core a small cone probe (0.95 mm; 30° cone semi-angle) attached to a thin metal shaft was penetrated to 15 mm depth from the soil surface with a rate equal to 4 mm min^−1^. Penetration resistance between 4.5 and 9.8 mm depth was subsequently averaged. On 17 May 2018 (22 DAS), field penetration resistance was tested in four locations per each plot penetrating a 12.83-mm-diameter cone probe (CP 40 Cone, Rimik Electronics, Toowoomba, AUSTRALIA) down in the soil up to a maximum depth of 0.5 m from the soil surface. Field penetration resistance is strongly affected by water potential, which averaged -5.9 ± 2.1 kPa in silt loam and -5.7 ± 2.0 kPa in sandy loam at 0.2 m depth.

On 27 August 2018 (124 DAS) soil cores were sampled at 0.1 m depth in plots planted with NRH and WT to assess soil hardness and modulus of elasticity by an indentation loading–unloading cycle (Naveed et al. 2018). After core equilibration at -10 kPa water potential on the suction ceramic plate, two indentation tests were performed on each soil core using a 3-mm-diameter spherical indenter fitted on the same universal testing frame and load cell used for measuring penetration resistance. During the test, the soil surface is indented at a rate of 1 mm min^−1^ rate to 0.8 – 1 mm depth until an impression is formed (*i.e*., loading) and then lifted up (unloading). The loading/un-loading vs displacement curve is then used to derive hardness and elasticity. Details of the indentation testing method and calculations for hardness and elasticity are provided in Naveed et al. (2018).

### Soil hydraulic properties

Soil hydraulic properties were measured on the same cores used for the indentation measurements, equilibrated at -10 kPa. Water sorptivity (*S*_W_), ethanol sorptivity (*S*_E_), and the water repellency index (R) were determined using a miniaturized infiltrometer device with a 0.4-mm tip radius. Details of the infiltrometer’s set up are provided in Hallett et al. (2003). The sorptivity describes the rate at which the soil absorbs the liquid (*i.e*., water or pure ethanol). Water sorptivity can be affected by soil water potential, particle hydrophobicity and both pore volume and structure. To isolate the effect of particle hydrophobicity, ethanol sorptivity was determined because its non-polar nature provides a reference of liquid transport that is not affected by hydrophobicity of the soil particles. Liquid absorption by the soil from the infiltrometer reservoir was recorded from a 0.1 mg balance at 1 s intervals. After about 30 s, the flow rate (*i.e*., weight drop of the infiltrometer reservoir), *Q*, was stable and hence used to calculate sorptivity (Eq. 2) following Leeds-Harrison et al. (1994).

2 $$S=\sqrt{\frac{Qf}{4br}}$$

where *f* is the fillable air-porosity, *b* a parameter that depends on the soil–water diffusivity function and *r* is the radius of the infiltrometer tip. *b* is in the range 0.5 < *b* < π/4. In the present study, the typical average value 0.55 was used.

The water repellency index was calculated from the ratio between the sorptivity of ethanol, *S*_*E*_, and water sorptivity, *S*_*W*_, (Eq. 3) in agreement with Tillman et al. (1989).

3 $$R=1.95\frac{{S}_{\mathrm{E}}}{{S}_{\mathrm{W}}}$$

An R value equal to 1 indicates a non-water-repellent soil (*i.e*., non-hydrophobic soil particles).

### Statistical analysis

Statistical analysis was performed using GenStat 19^th^ edition (VSN International), R version 4.0.0 (R Foundation for Statistical Computing, Vienna, Austria) and SigmaPlot14 (Systat Software Inc). Measurements that were repeated over time (*e.g*., PAW during the growing season) were analysed using the restricted maximum likelihood (REML) for repeated measurements. Differences between genotypes and soil textures for single time measurements (*e.g*., water sorptivity and repellency) were determined with two-way analysis of variance (ANOVA). Differences between genotypes within the same sampling and soil texture were assessed with one-way ANOVA, followed by post hoc Tukey’s test. Data that did not follow a normal distribution were log-transformed and checked again for normal distribution prior to ANOVA. The significance of correlations established in this study were tested by regression analyses. Results were considered statistically significant when *P* ≤ 0.05. Data variability is expressed as mean ± standard error of mean.

## Results

### Root growth and soil water retention properties over the growing season

**Fig. 1:**
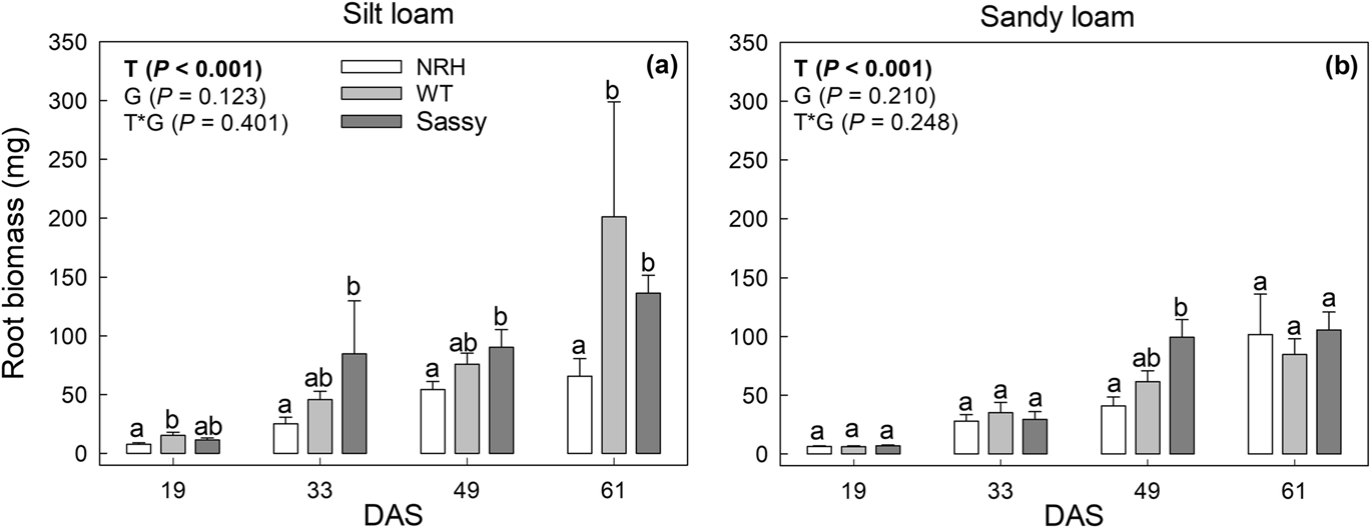
Variation in root biomass (mg) of contrasting root hair genotypes grown in the field in silt loam (a) and sandy loam (b) soils in 2018. Four sampling campaigns were carried out: 19, 33, 49 and 61 days after sowing (DAS). Data are the mean of eight replicates (2018), with error bars representing the s.e. Differences between genotypes over the growing season were established using the REML analysis, *P*-values are reported and significant (*P* < 0.05) parameters are in bold, with ‘T’ representing time, ‘G’ representing genotype and ‘G × T’ representing the interaction of genotype and time. Identical letters indicate no significant differences between genotypes within the same sampling campaign as tested using one-way ANOVA followed by a post-hoc Tukey’s test. NRH represents the no root hair genotype and WT the wildtype (cv Optic), along a separate cv Sassy

**Fig. 2:**
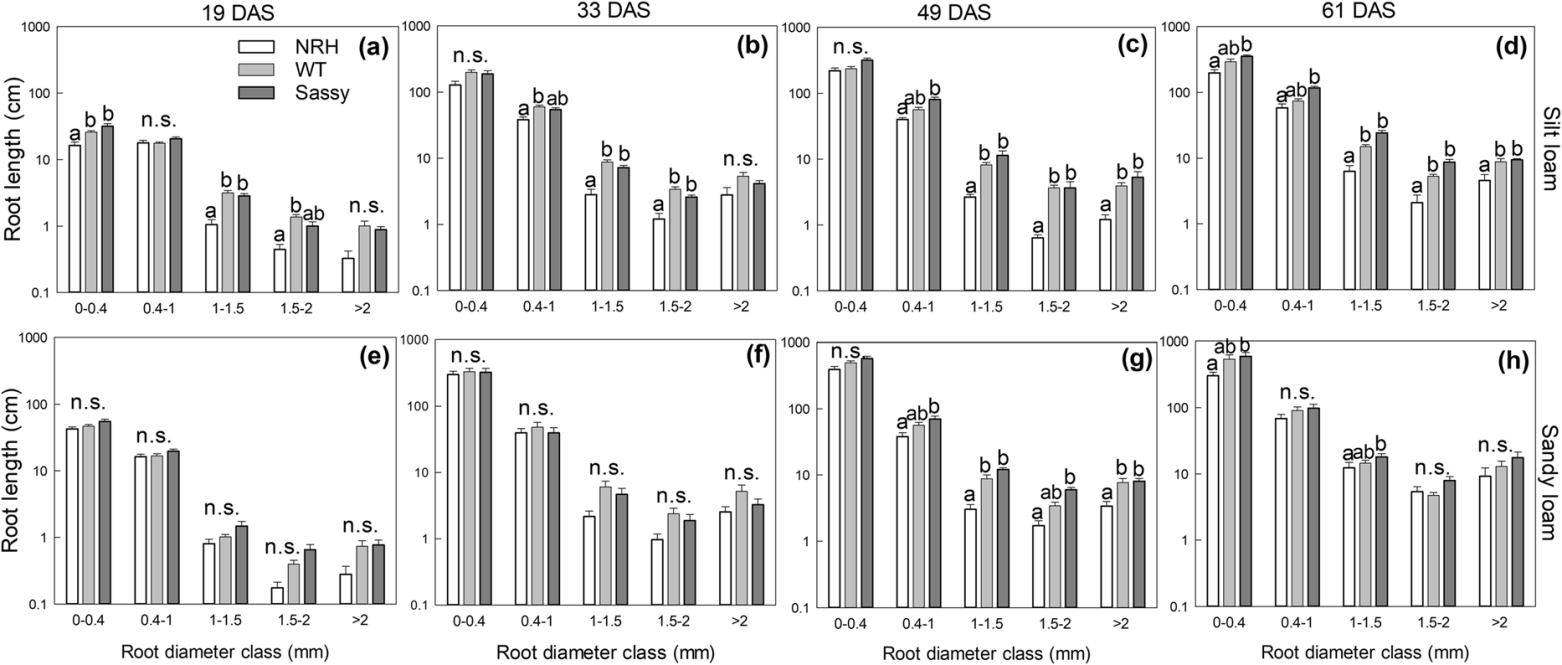
Average root length (cm) per diameter class (0–0.4 mm; 0.4–1 mm; 1–1.5 mm; 1.5–2 mm; > 2 mm) of contrasting root hair genotypes grown in the field in silt loam (a; b; c; d) and sandy loam (e; f; g; h) soils. Four sampling campaigns were carried out: 19 (a; c), 33 (b; f), 49 (c; g) and 61 (d; h) days after sowing (DAS). Data are the mean of eight replicates, with error bars representing the s.e. Identical letters indicate no significant differences between genotypes, as tested using one-way ANOVA followed by a post-hoc Tukey’s test. NRH represents the no root hair genotype and WT the wildtype (cv Optic), along a separate cv Sassy

The soil water retention properties showed expected differences between soil types, with changes also found as the growing season progressed. Root biomass increased from an average of 11.5 to 134.4 mg plant^−1^ in silt loam soil and from 6.5 to 97.3 mg plant^−1^ in sandy loam soil from 19 to 61 DAS, with significant differences found between genotypes in both soil textures (Fig. 1). For all genotypes and time points, most of root length was represented by fine roots, with diameters thinner than 0.4 mm (Fig. 2). Significant differences in root length between genotypes were mainly found in silt loam soil, with WT generally exhibiting greater root length than NRH for roots thicker than 1 mm. Significant differences in root length per diameter class between those two genotypes were generally absent in sandy loam soil, while in either soil NRH did not exhibit greater root length than WT for any of the diameter classes (Fig. 2).

Soil water retention properties varied between time points, with PAW increasing over the growing season (from 20 to 124 DAS) in sandy loam soil, from 0.24 to 0.29 m^3^ m^−3^ at 0.1 m depth (*P* < 0.001) and from 0.25 to 0.29 m^3^ m^−3^ at 0.2 m depth (*P* = 0.022; Table 1). In silt loam soil, PAW increased significantly (*P* = 0.022) from 0.21 to 0.23 m^3^ m^−3^ over the growing season at 0.1 m depth, while PAW did not differ significantly between 20 DAS (0.22 m^3^ m^−3^) and 124 DAS (0.21 m^3^ m^−3^) at 0.2 m depth. While following the first sampling, PAW was 14% greater in sandy loam compared to the silt loam at both 0.1 and 0.2 m depth; this difference increased at the end of the growing season to 26% at 0.1 m and 38% at 0.2 m. No significant differences were found in PAW between genotypes in either soil textures. Related parameters, such as porosity and air-capacity, reduced significantly at the end of the growing season in sandy loam soil (*P* < 0.001; Table 1). For instance, total porosity at 0.2 m decreased from 0.47 (20 DAS) to 0.39 m^3^ m^−3^ (124 DAS), while air-capacity decreased from 0.11 to 0.02 m^3^ m^−3^ over the same period (Table 1). Similar changes in time were also observed in terms of the water retained by soil at PWP (*i.e*., -1500 kPa; Table 1), which decreased significantly (*P* < 0.001 at 0.1 m; *P* = 0.02 at 0.2 m) as the season progressed (from 20 to 124 DAS) in sandy loam soil. The water content associated with PWP in sandy loam soil decreased from 0.16 to 0.12 m^3^ m^−3^ at 0.1 m, and from 0.15 to 0.12 m^3^ m^−3^ at 0.2 m. The water content in the silt loam soil at -1500 kPa decreased from 0.20 to 0.15 m^3^ m^−3^ at 0.1 m (*P* < 0.001), and from 0.21 to 0.17 m^3^ m^−3^ at 0.2 m (*P* = 0.053). Despite the general decrease of the water retained at PWP with the progress of the growing season, the silt loam was still able to hold 55% more non-usable water than the sandy loam. There were no significant differences between genotypes for any of the parameters described at any time point.

**Table 1:** Permanent wilting point (PWP), average plant available water (PAW), porosity and air capacity recorded in NRH, WT and SASSY plots in silt loam and sandy loam soils at 0.1 and 0.2 m depths during the growing season (*i.e*., days after sowing, DAS). Data are the mean of four replicates ± standard error of mean, with differences between genotypes, soil textures, soil depths and time of sampling established using REML for repeated measurements from which the *F* and *P*-value data are derived. Only significant (*P* ≤ 0.05) factors and their interactions, for any of the parameters considered, are reported. NRH represents the no root hair genotype and WT the wildtype (cv Optic), along a separate cv Sassy

DAS	Soil	Depth (m)	Genotype	PWP (m^3^m^−3^)		PAW (m^3^m^−3^)		Porosity (m^3^m^−3^)		Air capacity (m^3^m^−3^)	
19–20	Sandy loam	0.1	NRH	0.16 ± 0.01		0.25 ± 0.01		0.40 ± 0.01		0.03 ± 0.01	
			WT	0.16 ± 0.01		0.25 ± 0.00		0.40 ± 0.01		0.04 ± 0.01	
			Sassy	0.15 ± 0.01		0.23 ± 0.02		0.42 ± 0.02		0.06 ± 0.03	
		0.2	NRH	0.14 ± 0.02		0.22 ± 0.01		0.52 ± 0.04		0.15 ± 0.03	
			WT	0.15 ± 0.01		0.25 ± 0.01		0.36 ± 0.02		0.09 ± 0.01	
			Sassy	0.15 ± 0.00		0.27 ± 0.01		0.48 ± 0.03		0.10 ± 0.02	
	Silt loam	0.1	NRH	0.19 ± 0.01		0.22 ± 0.02		0.37 ± 0.00		0.05 ± 0.01	
			WT	0.20 ± 0.01		0.20 ± 0.01		0.39 ± 0.02		0.05 ± 0.01	
			Sassy	0.20 ± 0.02		0.21 ± 0.01		0.38 ± 0.03		0.03 ± 0.02	
		0.2	NRH	0.20 ± 0.03		0.21 ± 0.01		0.41 ± 0.04		0.05 ± 0.03	
			WT	0.20 ± 0.00		0.22 ± 0.01		0.40 ± 0.02		0.05 ± 0.01	
			Sassy	0.22 ± 0.01		0.22 ± 0.02		0.37 ± 0.01		0.03 ± 0.00	
50–51	Sandy loam	0.1	NRH	0.14 ± 0.01		0.27 ± 0.02		0.49 ± 0.02		0.08 ± 0.02	
			WT	0.14 ± 0.00		0.30 ± 0.01		0.47 ± 0.02		0.08 ± 0.02	
			Sassy	0.14 ± 0.01		0.28 ± 0.01		0.48 ± 0.02		0.08 ± 0.01	
		0.2	NRH	0.14 ± 0.01		0.28 ± 0.02		0.45 ± 0.03		0.13 ± 0.04	
			WT	0.13 ± 0.00		0.26 ± 0.03		0.48 ± 0.02		0.15 ± 0.04	
			Sassy	0.14 ± 0.01		0.28 ± 0.01		0.45 ± 0.03		0.11 ± 0.03	
	Silt loam	0.1	NRH	0.17 ± 0.00		0.23 ± 0.02		0.43 ± 0.02		0.09 ± 0.02	
			WT	0.19 ± 0.02		0.23 ± 0.02		0.44 ± 0.04		0.10 ± 0.03	
			Sassy	0.21 ± 0.01		0.22 ± 0.02		0.36 ± 0.03		0.05 ± 0.02	
		0.2	NRH	0.19 ± 0.01		0.25 ± 0.01		0.39 ± 0.02		0.07 ± 0.03	
			WT	0.20 ± 0.01		0.21 ± 0.03		0.35 ± 0.02		0.08 ± 0.03	
			Sassy	0.22 ± 0.03		0.23 ± 0.03		0.37 ± 0.03		0.06 ± 0.02	
124	Sandy loam	0.1	NRH	0.12 ± 0.00		0.29 ± 0.01		0.40 ± 0.01		0.02 ± 0.01	
			WT	0.12 ± 0.01		0.28 ± 0.02		0.39 ± 0.02		0.02 ± 0.01	
			Sassy	0.11 ± 0.00		0.30 ± 0.01		0.39 ± 0.01		0.01 ± 0.00	
		0.2	NRH	0.11 ± 0.01		0.29 ± 0.01		0.40 ± 0.03		0.04 ± 0.02	
			WT	0.11 ± 0.01		0.29 ± 0.01		0.36 ± 0.02		0.01 ± 0.00	
			Sassy	0.13 ± 0.01		0.28 ± 0.01		0.40 ± 0.01		0.02 ± 0.00	
	Silt loam	0.1	NRH	0.16 ± 0.01		0.23 ± 0.01		0.40 ± 0.01		0.03 ± 0.01	
			WT	0.15 ± 0.01		0.23 ± 0.01		0.40 ± 0.02		0.02 ± 0.00	
			Sassy	0.15 ± 0.00		0.24 ± 0.00		0.40 ± 0.01		0.03 ± 0.01	
		0.2	NRH	0.18 ± 0.01		0.22 ± 0.01		0.38 ± 0.00		0.01 ± 0.00	
			WT	0.18 ± 0.01		0.21 ± 0.01		0.40 ± 0.02		0.04 ± 0.01	
			Sassy	0.16 ± 0.02		0.20 ± 0.01		0.43 ± 0.04		0.06 ± 0.03	
*REML*				*F*	*P*	*F*	*P*	*F*	*P*	*F*	*P*
Time				25.34	< 0.001	11.01	< 0.001	6.60	0.002	29.10	< 0.001
Soil texture				201.5	< 0.001	80.22	< 0.001	32.11	< 0.001	7.43	0.010
Soil depth				1.45	0.236	0.64	0.428	0.08	0.777	8.65	0.006
Time × Soil texture				0.98	0.382	3.79	0.027	10.79	< 0.001	3.94	0.024
Time × Soil depth				0.33	0.723	1.75	0.181	6.08	0.004	1.21	0.305
Soil texture × Soil depth				6.18	0.018	0.01	0.913	2.63	0.114	11.63	0.002

The genotype and its interactions did not produce a significant difference for any of the parameters considered

**Fig. 3:**
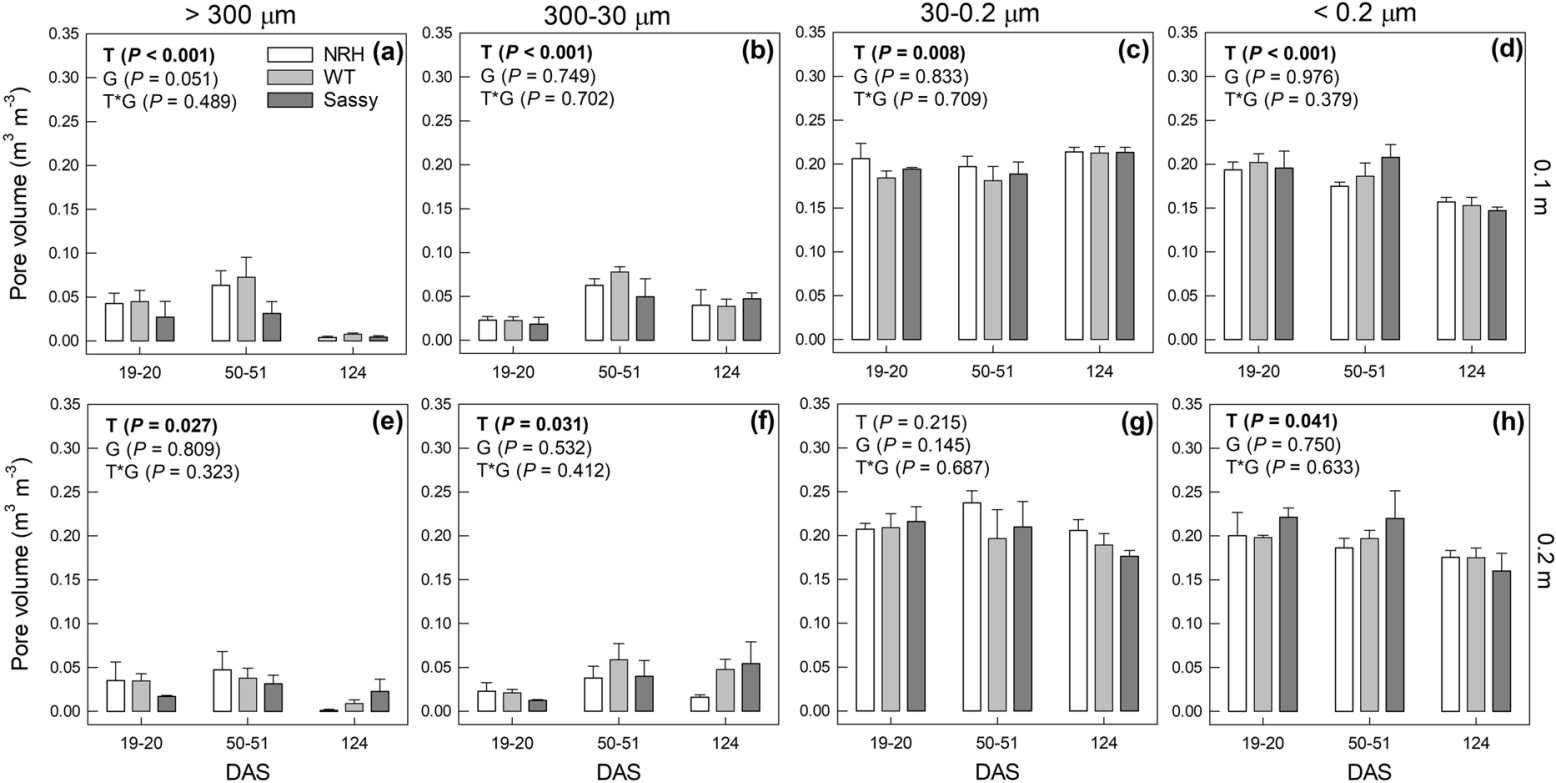
Estimated pore volume per diameter class: aeration pores (> 300 µm; a, e); drainage pores (300 – 30 µm; b, f); slow drainage and retention pores (30 – 0.2 µm; c, g); pores holding not-useful water for plants (< 0.2 µm; d, h) for cores sampled from NRH, WT and Sassy plots in silt loam soil at 0.1 m (a, b, c, d) and 0.2 m (a, f, g, h) depths during the growing season (*i.e*., days after sowing, DAS). The total volume of the soil core was equal to 95 cm.^3^. Data are the mean of four replicates ± standard error of mean. Differences between genotypes over the growing season were established using the REML analysis, *P*-values are reported and significant (*P* < 0.05) parameters are in bold, with ‘T’ representing time, ‘G’ representing genotype and ‘G × T’ representing the interaction of genotype and time. NRH represents the no root hair genotype and WT the wildtype (cv Optic), along a separate cv Sassy

**Fig. 4:**
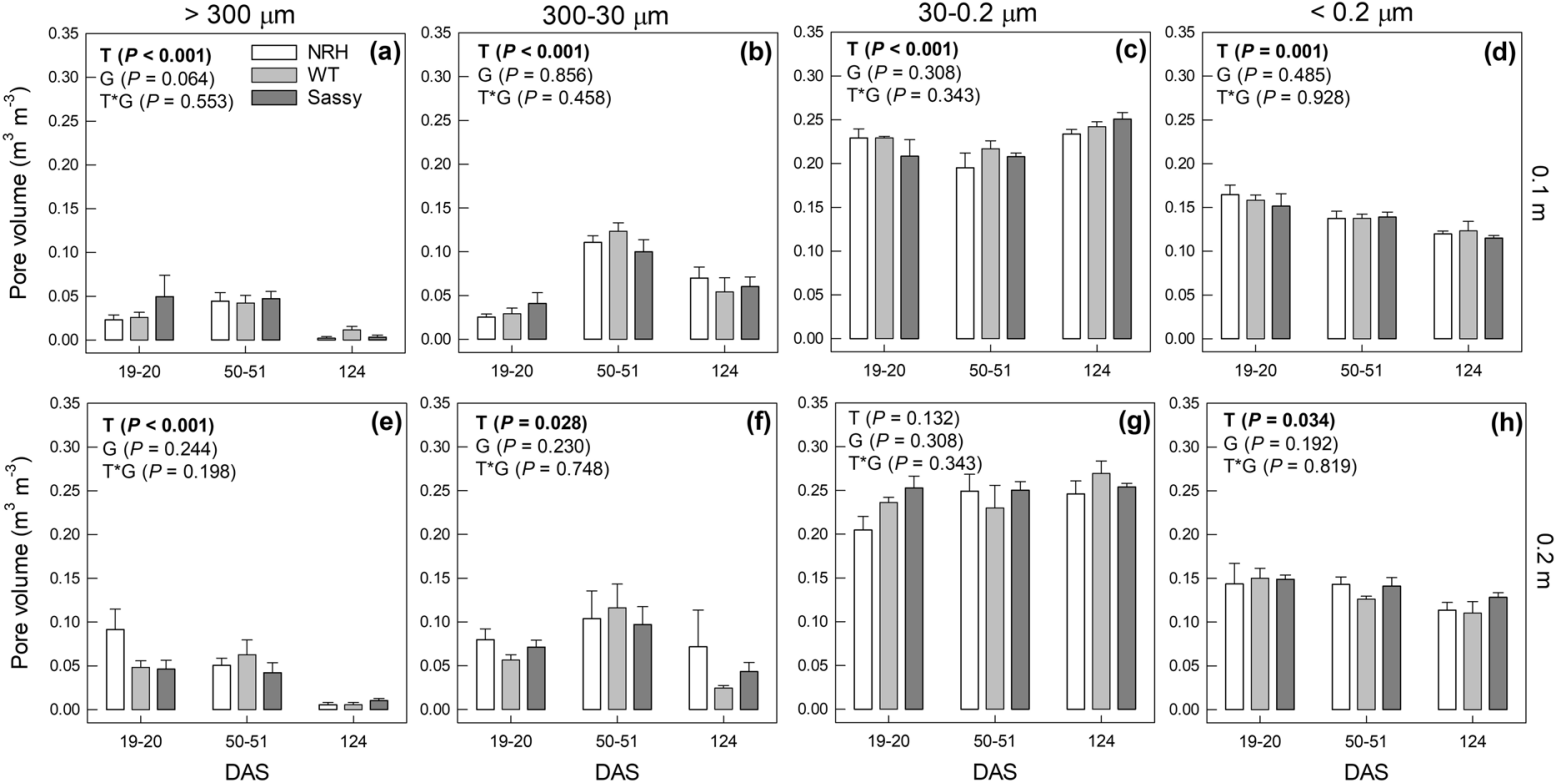
Estimated pore volume per diameter class: aeration pores (> 300 µm; a, e); drainage pores (300 – 30 µm; b, f); slow drainage and retention pores (30 – 0.2 µm; c, g); pores holding not-useful water for plants (< 0.2 µm; d, h) for cores sampled from NRH, WT and Sassy plots in sandy loam soil at 0.1 m (a, b, c, d) and 0.2 m (a, f, g, h) depths during the growing season (*i.e*., days after sowing, DAS). The total volume of the soil core was equal to 95 cm.^3^. Data are the mean of four replicates ± standard error of mean. Differences between genotypes over the growing season were established using the REML analysis, *P*-values are reported and significant (*P* < 0.05) parameters are in bold, with ‘T’ representing time, ‘G’ representing genotype and ‘G × T’ representing the interaction of genotype and time. NRH represents the no root hair genotype and WT the wildtype (cv Optic), along a separate cv Sassy

Over the growing season there was a re-arrangement of the pore size distribution for silt loam (Fig. 3) and sandy loam (Fig. 4) soils. The volume of macro-pores (*d* > 300 µm), responsible for soil aeration, decreased significantly (*P* < 0.05) in both soil types and depths as the season progressed, with an average volume loss at 124 DAS ranging between 42 and 85% of the value recorded at 20 DAS. While the aeration pores’ volume shrank (*d* > 300 µm) in both soils and depths, the volume of drainage pores (*d* = 30 – 300 µm) in the surface soil (*i.e.*, 0.10 m) increased by 156 and 133% over the growing season in silt loam (Fig. 3) and sandy loam (Fig. 4), respectively. In contrast, the volume of drainage pores in sandy loam soil, sampled at 0.2 m, decreased by 37% over the same period (*P* = 0.009; Fig. 4). The volume occupied by micropores (*d* < 0.2 µm) in surface soil showed a significant (*P* < 0.001) and consistent reduction in both silt loam (-22%; Fig. 3) and sandy loam (-24%; Fig. 4) soils, while at the same sampling depth the volume of pores responsible for slow water drainage and retention of PAW (*d* = 0.2 – 30 µm) increased significantly in both soil types (+ 11%, *P* ≤ 0.001). No significant change along the growing season was found in deeper soil in the volume of pores with diameters ranging between 0.2 and 30 µm. There was no significant impact of genotype on pore size distribution in either soil or time point.

### Soil hydraulic properties

**Fig. 5:**
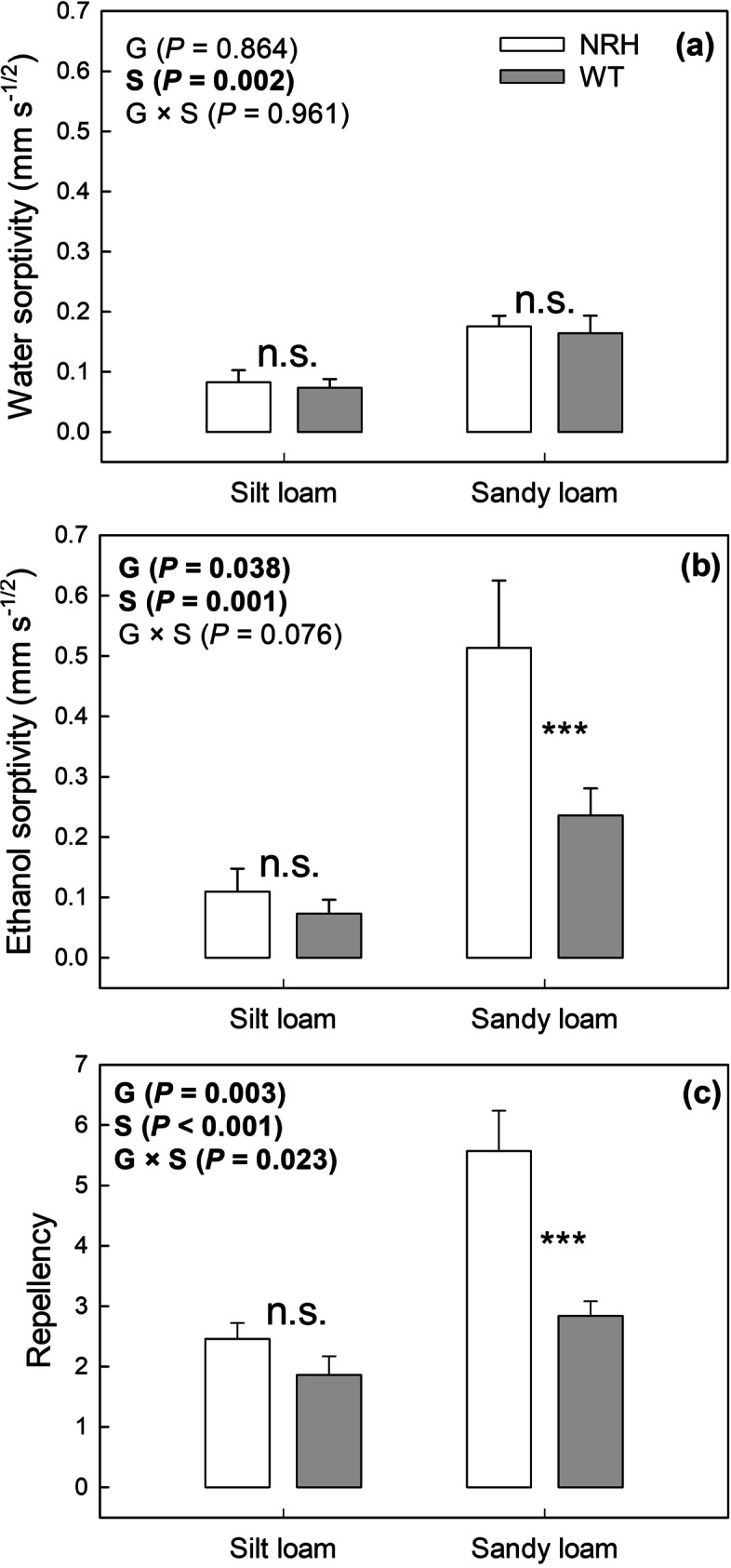
Water sorptivity (a), ethanol sorptivity (b) and repellency (c) measured in soil cores sampled from different experimental treatments (*i.e.*, NRH and WT) in both silt and sandy loam fields. Data are the mean of eight replicates, with error bars representing the s.e. Differences between genotypes and soil textures were established using two-way ANOVA, *P*-values are reported and significant (*P* < 0.05) parameters are in bold, with ‘G’ representing genotype, ‘S’ representing soil texture, and ‘G × S’ representing the interaction of genotype and soil texture. *** indicates a significant difference between NRH and WT samples, while n.s. indicates a lack of significant differences. NRH represents the no root hair genotype and WT the wildtype (cv Optic)

Samples collected from the silt loam field had significantly smaller water (*P* = 0.002) and ethanol (*P* = 0.001) sorptivity than those collected from sandy loam plots (Fig. 5). Overall silt loam samples showed an average 54 and 74% smaller water and ethanol sorptivity, respectively, compared to sandy loam samples. Similarly, silt loam samples were 46% less repellent to water than sandy loam samples (Fig. 5c). Ethanol sorptivity was 17 and 96% greater than water sorptivity in silt loam and sandy loam, respectively. While there were no significant differences in water sorptivity between genotypes in either soil, ethanol sorptivity was significantly smaller (*P* = 0.044) in WT (0.24 mm s^−1/2^) compared to NRH (0.52 mm s^−1/2^) samples in sandy loam soil. Within the same soil texture, differences in water repellency between genotypes were even more significant (*P* = 0.007), being 49% smaller in WT than NRH samples. No significant differences between genotypes for either variable were found in silt loam soil, although the same trends were observed.

### Soil mechanical properties

**Fig. 6:**
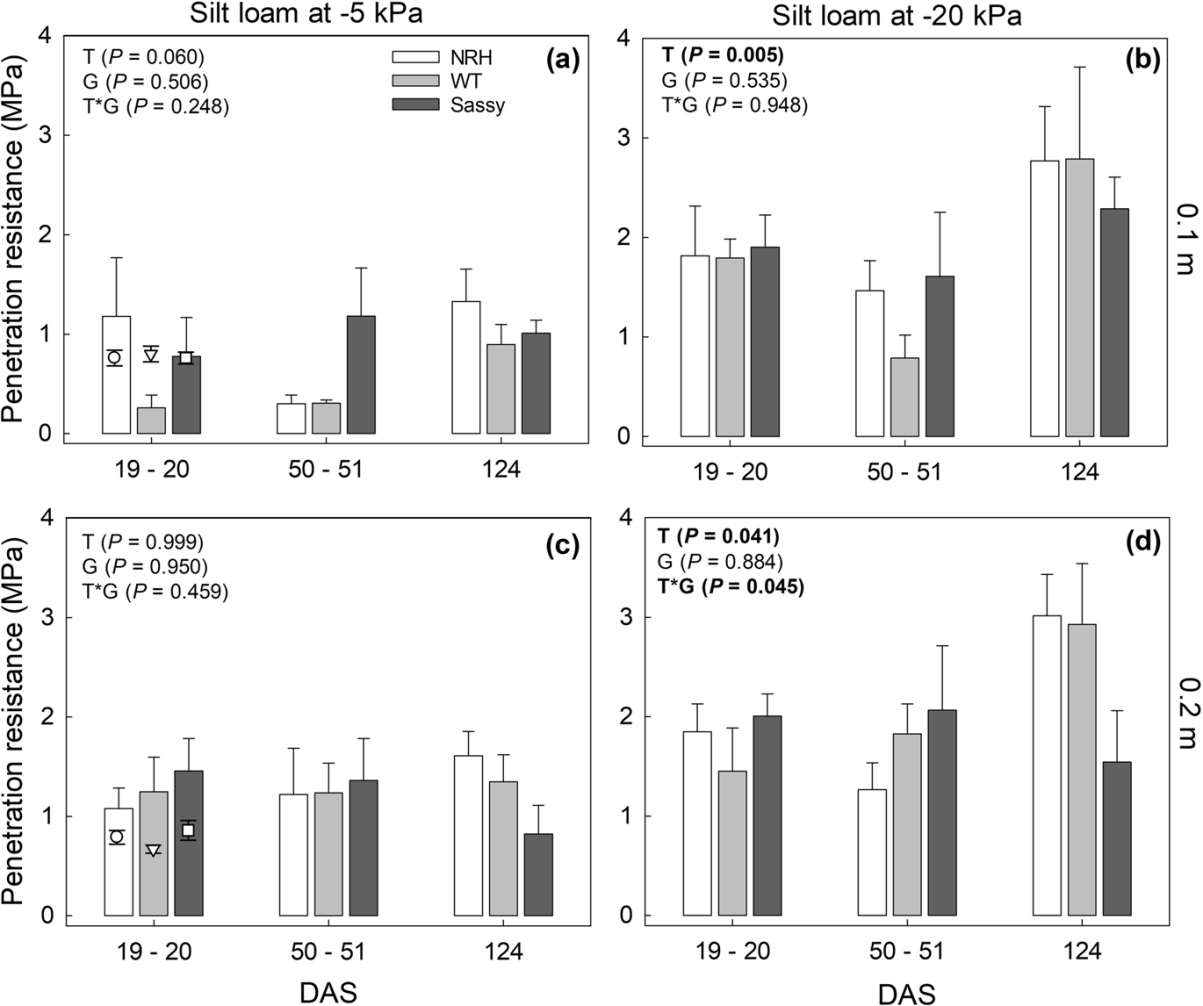
Average penetration resistance measured in soil cores sampled in NRH, WT and Sassy plots at 0.1 m (a; b) and 0.2 m (c; d) in the silt loam field during the growing season, and equilibrated on a suction ceramic plate at -5 kPa (a; c) and -20 kPa (b; d). Data are the mean of four replicates, with error bars representing the s.e. Differences between genotypes along the growing season were established using the REML analysis, *P*-values are reported and significant (*P* < 0.05) parameters are in bold, with ‘G’ representing genotype, ‘T’ representing time, and ‘G × T’ representing the interaction of genotype and time. The scatter plot indicates the penetration resistance measured in the field in May 2018 at 0.1 and 0.2 m depth in NRH (○), WT () and SASSY (□) plots using a field penetrometer. The soil water potential in the field averaged -5.9 ± 2.1 kPa at 0.2 m depth. Data are the mean of four replicates with error bars representing the s.e. NRH represents the no root hair genotype and WT the wildtype (cv Optic), along a separate cv Sassy

**Fig. 7:**
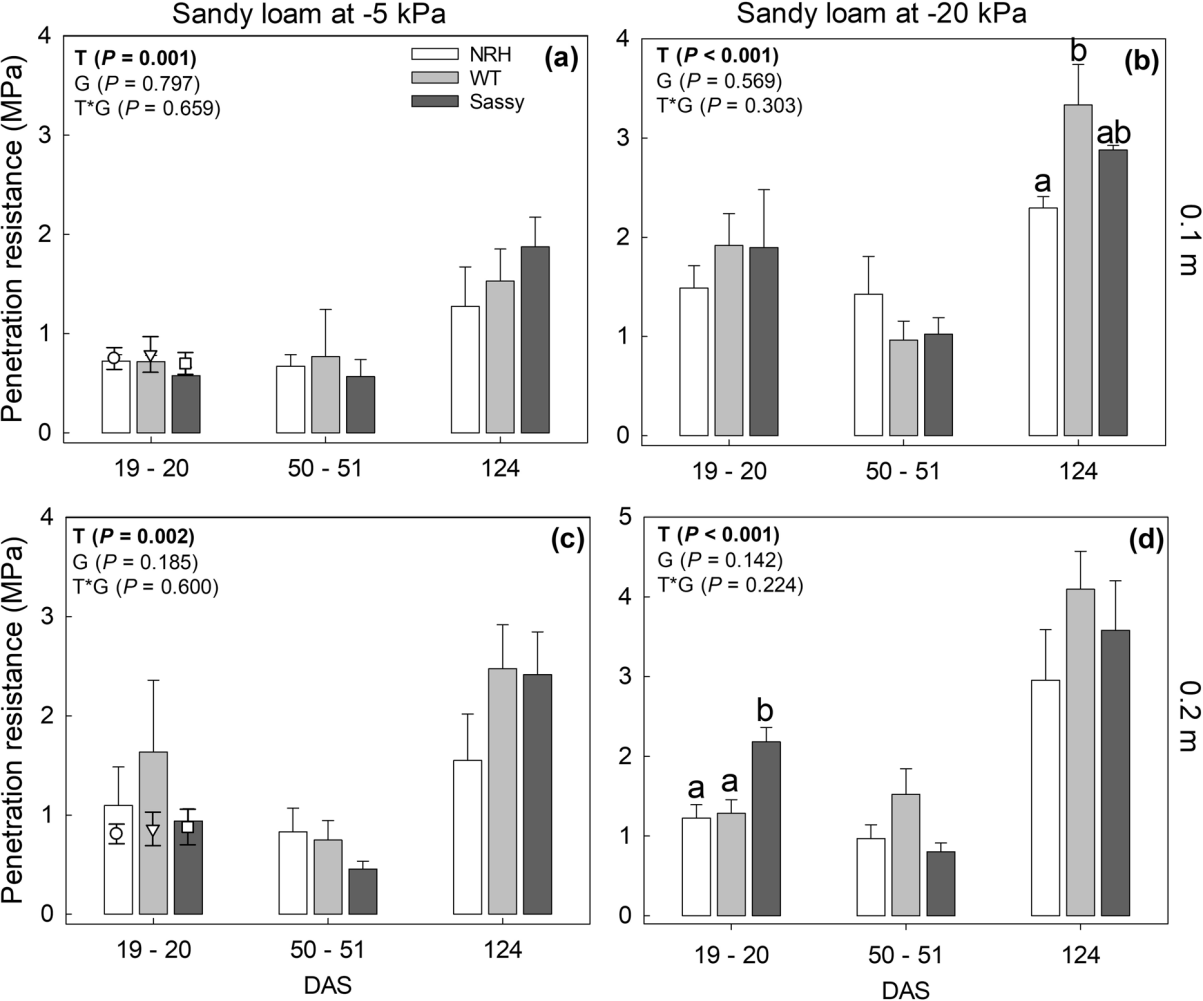
Average penetration resistance measured in soil cores sampled in NRH, WT and Sassy plots at 0.1 m (a; b) and 0.2 m (c; d) in the sandy loam field during the growing season, and equilibrated on a suction ceramic plate at -5 kPa (a; c) and -20 kPa (b; d). Data are the mean of four replicates, with error bars representing the s.e. Differences between genotypes along the growing season were established using the REML analysis, *P*-values are reported and significant (*P* < 0.05) parameters are in bold, with ‘G’ representing genotype, ‘T’ representing time, and ‘G × T’ representing the interaction of genotype and time. The scatter plot indicates the penetration resistance measured in the field in May 2018 at 0.1 and 0.2 m depth in NRH (○), WT () and SASSY (□) plots using a field penetrometer. The soil water potential in the field averaged -5.7 ± 2.0 kPa at 0.2 m depth. Data are the mean of four replicates with error bars representing the s.e. Identical letters indicate no significant differences between genotypes for each sampling campaign, as tested using one-way ANOVA followed by a post-hoc Tukey’s test. NRH represents the no root hair genotype and WT the wildtype (cv Optic), along a separate cv Sassy

Penetration resistance (PR) was measured for the same samples tested for soil water retention properties and was significantly affected by sampling depth (*P* = 0.008), sampling period (*i.e.*, time after sowing; *P* < 0.001) as well as soil drying (*P* < 0.001; Figs. 6 and 7). The penetration resistance measured in the laboratory with a 0.95 mm diameter cone probe was consistent with the data collected in the field using a 12.83 mm diameter cone over the same period (Figs. 6 and 7). In the silt loam field, penetration resistance ranged between 0.31 and 1.94 MPa at 0.1 m, and between 0.34 and 2.02 MPa at 0.2 m (Fig. 6). Similarly, PR measured in the laboratory, on cores collected in the field at 20 DAS and equilibrated to -5 kPa, varied between 0.34 and 2.75 MPa at 0.1 m depth, and between 0.52 and 2.36 MPa at 0.2 m depth. Following equilibration at -20 kPa (*i.e.*, after drying), the silt loam soil had a significant strength gain of 0.5 MPa (*i.e*., + 40%) at both depths (Fig. 6). The water potential in the silt loam field at 27 DAS ranged between -4 and -20 kPa (data not shown). A similar relationship between the PR measured in the lab and that assessed in the field was found for the sandy loam soil (Fig. 7), with no significant differences found between the two soil types. However, samples collected in sandy loam soil had a different strength gain with drying (*i.e.*, water potential dropping from -5 to -20 kPa) between the two testing depths, with the penetration resistance increasing by 1.1 MPa (*i.e.*, + 160%) at 0.1 m depth, compared to the 0.3 MPa (*i.e.*, + 28%) strength gain at 0.2 m. The soil water potential recorded in the sandy loam field at the time of the PR tests varied between -18 and -36 kPa (data not shown). Penetration resistance showed a significant interaction (*P* = 0.020) between soil texture, sampling date (*i.e*., days after sowing) and depth. Early in the growing season (20 DAS) the PR in sandy loam samples, collected at 0.2 m in SASSY plots (2.18 ± 0.18 MPa), was significantly greater (*P* = 0.006) than that measured in NRH (1.23 ± 0.17 MPa) and WT (1.29 ± 0.17 MPa) plots (cores equilibrated at -20 kPa; Fig. 7d). In contrast, at the end of the growing season (124 DAS) PR was significantly (*P* = 0.039) greater in WT plots (3.33 ± 0.41 MPa) than in NRH (2.29 ± 0.11 MPa) plots in sandy loam soil at 0.1 m (cores equilibrated at -20 kPa; Fig. 7b). SASSY plots (2.88 ± 0.05 MPa) had intermediate PR values and did not significantly differ from WT or NRH.

**Fig. 8:**
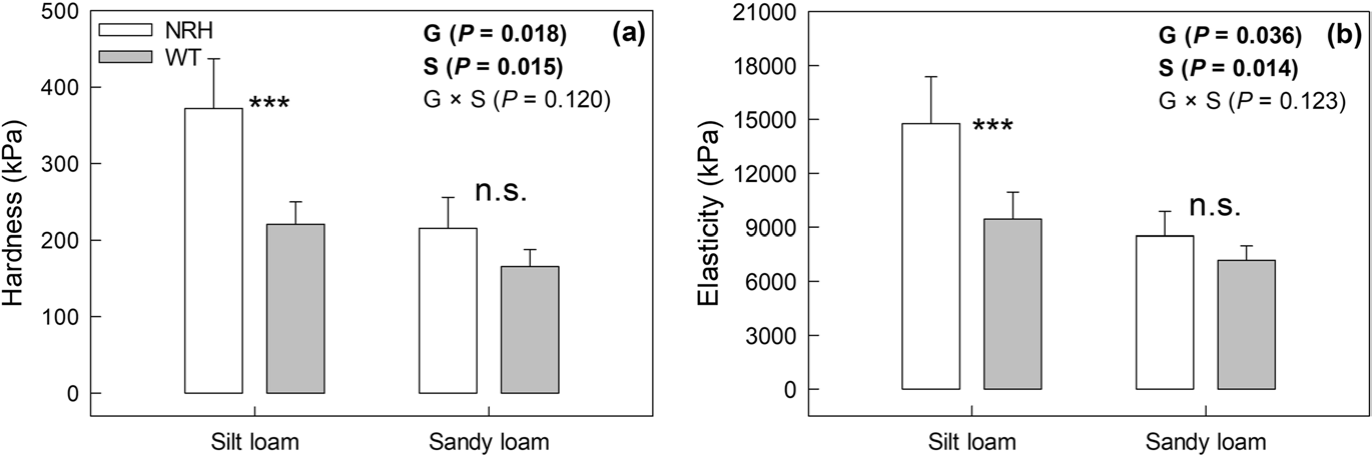
Hardness (a) and elasticity (b) measured in soil cores sampled from different experimental treatments (*i.e.*, NRH and WT) in both silt and sandy loam fields. Data are the mean of eight replicates, with error bars representing the s.e. Differences between genotypes and soil textures were established using two-way ANOVA, *P*-values are reported and significant (*P* < 0.05) parameters are in bold, with ‘G’ representing genotype, ‘S’ representing soil texture, and ‘G × S’ representing the interaction of genotype and soil texture. *** indicates a significant difference between NRH and WT samples, while n.s. indicates a lack of significant differences. NRH represents the no root hair genotype and WT the wildtype (cv Optic)

The hardness (H) and elasticity (E) measured from the unloading of the spherical indenter differed significantly between soil textures (*P* = 0.014), with the silt loam exhibiting greater hardness (+ 74%) and elasticity (+ 73%) compared to sandy loam (Fig. 8). More interestingly, there was a significant effect of the barley genotype on the mechanical properties of soil in field conditions, with the presence of root hairs significantly affecting both soil hardness (*P* = 0.031) and elasticity (*P* = 0.048) in silt loam soil (Fig. 8). Indeed, soil hardness decreased by 50% in the presence of root hairs, while soil elasticity was reduced by 36%. In sandy loam soil, we did not find significant differences between genotypes, however we observed the same trend as in silt loam soil.

## Discussion

Many biophysical processes driving rhizosphere dynamics have been identified in laboratory conditions (Bengough 2012), but despite the role of roots as soil engineers being advocated for regenerative agriculture (Jin et al. 2017), the importance of these processes under field conditions has not been validated. In particular, the biophysical effects of root hairs on soil physical properties have been investigated mainly under artificial conditions (Koebernick et al. 2017, 2019). Therefore, the results gathered in this study represent the first effort to translate rhizosphere-scale processes to full-scale field conditions under natural climate and soil variability. Specifically, the presence of root hairs decreased soil hardness and elasticity, as well as water repellency but its significance was dependent on soil texture and had limited impact on some of the soil physical and water release parameters measured.

### Dynamics of soil physical properties during a growing season

The soil physical properties changed over time during the growing season, highlighting a reorganisation of the soil structure following ploughing and seed bed preparation. The air-capacity of the soil decreased significantly over time, with the estimated value of large pores, responsible of soil aeration, showing an abrupt drop at the end of the monitoring period (i.e., 124 days after sowing). This loss of macropores (> 300 µm) over the growing season is in agreement with previous studies, showing decreased aggregate size and closer aggregate packing (Hall 1993). The highlighted changes could have been driven by the dispersal of large tillage produced aggregates during rain events and plant rearrangement of soil particles. Fine soil particles from the aggregate outer skin are indeed dispersed under wetting, potentially clogging pores. At the same time, the fibrous roots of barley might have occupied macropores or contributed to closer soil packing (i.e., densification of the rhizosphere) by root radial growth and soil penetration (Bruand et al. 1996). Furthermore, wetting–drying cycles driven by evapotranspiration might have induced compressive forces and hence macropores shrinkage (Jin et al. 2017). It should be noted that the abrupt decrease of macropores was recorded at both depths and soil textures.

Soils’ ability to retain water changed over time, however this was dependent on soil texture and sampling depth. For instance, field capacity changed significantly over time only in silt loam soil at 0.2 m depth. The theoretical water available for plants was significantly affected by both time and soil texture. Similarly, as already discussed for air capacity, water retention properties might have been affected by pore rearrangement. While retention pores (30 – 0.2 µm) increased over time, there was a general decrease in the volume of pores retaining non available water (< 0.2 µm; Figs. 3 and 4) measured from the PWP at -1500 kPa. On a similarly structured soil near to this field experiment, a decrease in < 0.2 µm was observed over the growing season (Geris et al. 2021), despite a widespread assumption that these pores are affected by texture and organic matter so remain relatively static. This could be explained by the physical rearrangement of smaller soil pores to form mesopores, combined with the chemical properties of root exudates (Read et al. 2003). Since the volume of roots increased as the growing season progressed, it can also be hypothesised that, at 124 DAS, the loss of water stored in the roots played an increased influence on the overall water release curve of rooted soil. This effect would have been more remarkable at lower soil water potentials, when roots lose water and shrink (Lemcoff et al. 2006). Carminati et al. (2010) showed distinct water retention properties of the rhizosphere soil, that changed with root ageing and facilitated plant water uptake (Carminati and Vetterlein 2013). Therefore, we can hypothesise that soil alteration at the rhizosphere scale can also influence the water retention properties of bulk soil.

Results of the present study show the remarkable dynamics of soil physical properties over a relatively short period (i.e., one growing season). Therefore, when modelling or accounting for soil properties in the context of fundamental ecosystem services, such as plant growth, flood mitigation, water purification or carbon sequestration, particular attention should be paid to temporal dynamics instead of considering soil physical properties as fixed characteristics. Indeed, changes over the growing season in water retention, aeration and penetration resistance may have large impacts on plant growth and carbon sequestration processes. Generally fixed values are used to report these properties of soil based on one sampling time. These results could be therefore considered to develop empirical aging of soil physical parameters over the course of seasons.

### The biophysical effects of root hairs on soil in the field

The results of this study indicate that genotypes of barley differing in root hair phenotypes can significantly impact some soil physical properties. The sandy loam soil planted with the barley wild type showed 49% less water repellency compared with the soil planted with its hairless mutant. Although the mechanism inducing the smaller water repellency in the presence of the wild type has not been identified, it is possible to hypothesise that root hairs enhance the diffusion of root exudates in the rhizosphere (Holz et al. 2020). In laboratory studies, barley-root exudates have been shown to have a negligible effect on water repellency compared to maize-root exudates and chia-seeds mucilage due to their contrasting chemical composition (Naveed et al. 2019). Indeed, barley exudates have a large concentration of organic acids and a relatively small concentration in sugars, which generally determine soil hydrophobicity (Ahmed et al. 2016; Naveed et al. 2019; Zickenrott et al. 2016). Barley exudates can therefore act as surfactants, decreasing the surface tension of water (Naveed et al. 2019; Read et al. 2003) and hence soil water repellency (Ogunmokun et al. 2020). Furthermore, it may be hypothesised that the greater water stress, experienced by the hairless mutant (Marin et al. 2021), might have induced changes in the chemistry of root exudates to compensate for the disadvantage associated with the lack of root hairs (i.e., lower water and phosphorus uptake; Williams and de Vries (2020)). It has been demonstrated that mucilage-induced repellency can protect root tissues from water loss under drought, by temporarily disconnecting the root from dry soil (Carminati et al. 2010; Carminati and Vetterlein 2013), which incidentally appears to be the mechanism used by maize (Naveed et al. 2017), a species with much shorter and fewer root hairs (Brown et al. 2017).

In addition to the hydrological alterations, the presence of root hairs decreased both soil hardness (-50%) and elasticity (-36%). These mechanical changes can be explained by root hairs’ manipulation of the soil structure through physical intrusion (Koebernick et al. 2017) and exudate diffusion in the soil mass (Naveed et al. 2017). Koebernick et al. (2017) found a significantly greater pore volume fraction in the rhizosphere of the root-hair-bearing genotype imaged by computed tomography using synchrotron radiation. Furthermore, Naveed et al. (2017) showed that soil amended with barley root-exudates had weaker soil particle bonds, which was hypothesised to be functional to nutrient release and root foraging. However, a significantly smaller penetration resistance was recorded in soil cropped with the barley hairless mutant, which seems to contradict the soil hardness data. However, the specific parameters measured by these methods differ largely. While soil hardness was measured up to 1 mm depth using a spherical indenter, the penetration resistance was quantified by pushing a cone probe through 15 mm of soil. The indenter is almost strictly a spherical load that compresses the soil. The cone probe is likely a combination of compression and tension. Previous studies on plant traits associated with changes in the soil have focussed on contrasting species and root systems under controlled conditions (e.g., coarse *vs* fine (Bodner et al. 2014) or fibrous *vs* woody (Leung et al. 2018)), while our study is based on different genotypes of the same species (*Hordeum vulgare*) grown under field conditions and common agronomic practice, where laboratory-measured phenomena can often be easily hidden by soil variability. Furthermore, the observed differences were recorded following just one growing season on a soil that was previously disturbed by tillage and seed bed preparation. Although root traits and in particular root-soil interface traits are gathering growing interest (Hallett et al. 2022), there is still a lack of understanding on the relation between different root traits (including root hairs) and potential compensation mechanisms, particularly when different soils are considered (Vetterlein et al. 2022). In the present study, the main difference between genotypes was given by the presence/absence of root hairs, and although it is not possible to exclude a combined influence of different root traits as well as their interactions, this is not evident in the present study. Future work is needed to understand the relations between root traits and their effect on soil–plant interactions.

Soil–plant interaction is clearly a two-way relationship, where soil affects plant development and vice versa but despite this, research has mainly focussed on one direction of this relationship: the influence of soil on plant growth and productivity. Although the role of roots in shaping soil structure and hence soil physical properties is well recognised (Benard et al. 2016; Jin et al. 2017; Naveed et al. 2018; Tisdall and Oades 1979), the possibility to engineer soil in agroecosystems is still lacking mechanistic understanding, in particular when considering different varieties of cash-crops. Indeed, while several species of cover and break crops have been selected for specific root traits (e.g., large taproot penetrating compacted soil) and commercialised for soil improvements (e.g., tiller radish), the opportunity to choose specific varieties (i.e., genotypes) of cash crops to engineer soil properties has not been investigated and is urgently needed to solve the dichotomy between soil improvement and food production, using new tools for the sustainable management of agroecosystems. Our results indicate that the breeding of cash crops can and must also account for soil improvement in addition to yield, efficient use of resources and resilience to climate change and food insecurity (Ceccarelli and Grando 2020; Ceccarelli et al. 2010; Gregory and George 2011; Lynch 2007). This is a necessary step to shift agricultural production from the major driver of soil degradation (Gomiero 2016) to the paradigm of regenerative agriculture, which promotes soil-health regeneration and sustainability, and is driving recent national policy, rewarding farmers investing in soil health (European Commission 2020).

## Supplementary Information

Below is the link to the electronic supplementary material.

Supplementary file1 Suppl. Fig. 1 Soil texture analysis for (a) sandy loam sampled at 0-13 cm; (b) sandy loam sampled at 14-27 cm; (c) silt loam sampled at 0-13 cm; (d) silt loam sampled at 14-27 cm. Data are the mean of three replicates, with error bars representing the s.e. The USDA soil classification system was used for texture analysis, which was determined with the combination of wet sieving and laser diffraction (for particles smaller than 0.250 mm) (JPG 5346 KB)
