# Supplementary figures and images for: Impact of root hairs on microscale soil physical properties in the field
Source: Plant Soil. 2022 Jun 11;476(1-2):491–509. doi: 10.1007/s11104-022-05530-1 (PMC9381483; doi:10.1007/s11104-022-05530-1)

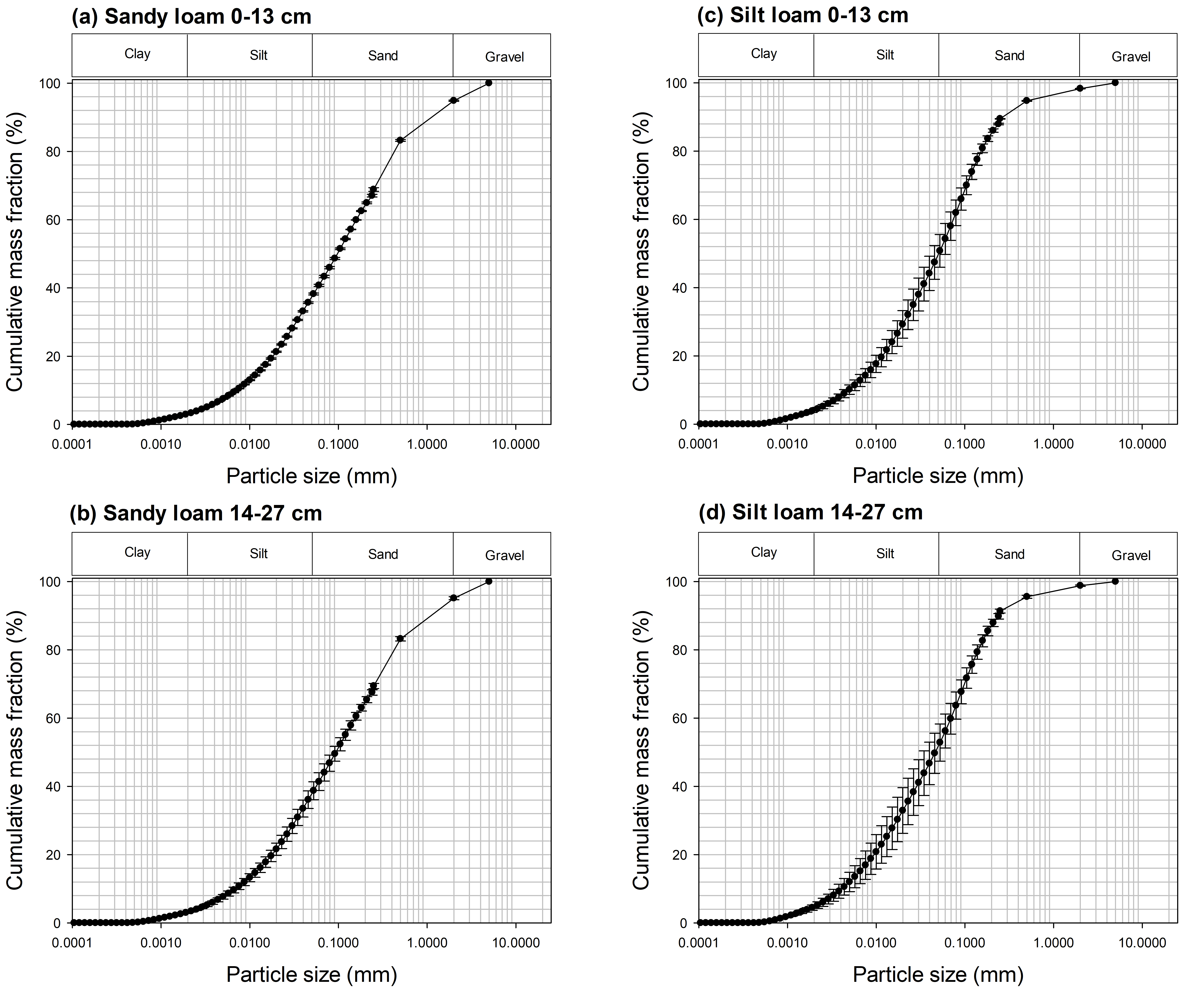

Supplement: Supplementary file 1 — Supplementary file1 Suppl. Fig. 1 Soil texture analysis for (a) sandy loam sampled at 0-13 cm; (b) sandy loam sampled at 14-27 cm; (c) silt loam sampled at 0-13 cm; (d) silt loam sampled at 14-27 cm. Data are the mean of three replicates, with error bars representing the s.e. The USDA soil classification system was used for texture analysis, which was determined with the combination of wet sieving and laser diffraction (for particles smaller than 0.250 mm) (JPG 5346 KB) [file 11104_2022_5530_MOESM1_ESM.jpg]
